# Potential of computational models in personalized treatment of obstructive sleep apnea: a patient-specific partial 3D finite element study

**DOI:** 10.1007/s10237-023-01788-1

**Published:** 2023-11-17

**Authors:** Venkat Ayyalasomayajula, Mads Moxness, Bjørn Skallerud

**Affiliations:** 1 Department of Structural Engineering, Norwegian University of Science and Technology, Trondheim, Norway; 2 Department of Otolaryngology, Aleris Hospital, Trondheim, Norway; 3 Department of Neuroscience, Norwegian University of Science and Technology, Trondheim, Norway

**Keywords:** Obstructive sleep apnea, Patient-specific modeling, UPPP, Palatal implants

## Abstract

The upper airway experiences mechanical loads during breathing. Obstructive sleep apnea is a very common sleep disorder, in which the normal function of the airway is compromised, enabling its collapse. Its treatment remains unsatisfactory with variable efficacy in the case of many surgeries. Finite element models of the upper airway to simulate the effects of various anatomic and physiologic manipulations on its mechanics could be helpful in predicting surgical success. Partial 3D finite element models based on patient-specific CT-scans were undertaken in a pilot study of 5 OSA patients. Upper airway soft tissues including the soft palate, hard palate, tongue, and pharyngeal wall were segmented around the midsagittal plane up to a width of 2.5 cm in the lateral direction. Simulations of surgical interventions such as Uvulopalatopharyngoplasty (UPPP), maxillo-mandibular advancement (MMA), palatal implants, and tongue implants have been performed. Our results showed that maxillo-mandibular advancement (MMA) surgery of 1 cm improved the critical closing pressure by at least 212.2%. Following MMA, the best improvement was seen via uvulopalatopharyngoplasty (UPPP), with an improvement of at least 19.12%. Palatal and tongue implants also offered a certain degree of improvement. Further, we observed possible interacting mechanisms that suggested simultaneous implementation of UPPP and tongue stiffening; and palatal and tongue stiffening could be beneficial. Our results suggest that computational modeling is a useful tool for analyzing the influence of anatomic and physiological manipulations on upper airway mechanics. The goal of personalized treatment in the case of OSA could be achieved with the use of computational modeling.

## Introduction

Obstructive sleep apnea (OSA) is a sleep disorder caused by a partial (hypopnea) or a complete (apnea) collapse of the upper airway during sleep (Maspero et al. 2015). Clinical manifestations of OSA are night snoring, headache when the patient wakes up, day-time sleepiness and a decline in cognitive performance Supriyatno et al. (2010), a 2 to 3-fold increased risk of cardiovascular and metabolic disease (Gottlieb and Punjabi 2020).

OSA is often attributed to a combination of anatomical malformations and non-anatomical causes (Eckert 2018; Neelapu et al. 2017). Anatomical causes include a narrow pharynx and constricted craniofacial structure. Non-anatomical contributors are caused by dysfunction in neuromuscular control and include impaired pharyngeal dilator muscle function, unstable control of breathing, and degradation of soft tissue mechanical function (Eckert et al. 2013).

Conservative treatment methods consist of behavioral measures such as abstinence from alcohol, avoiding supine sleep position, regular aerobic exercise, weight loss (Ashrafian et al. 2015), and the use of positive airway pressure devices (CPAP) (Qaseem et al. 2013) and mandibular repositioning devices (Ramar et al. 2015). However, long-term compliance is often a problem with these devices (Kribbs et al. 2012).

Surgical intervention is recommended only for select patients with moderate to severe OSA. The most extensively studied surgical procedure is uvulopalatopharyngoplasty (UPPP), which involves the resection of the uvula and part of the soft palate (Sheen and Abdulateef 2021). Maxillo- mandibular advancement surgery (MMA) on the other hand re-positions the bones of the upper and lower jaw to relieve airway obstruction (Prinsell 2002). Likewise, the most studied surgery targeting dilator muscle tone is hypoglossal nerve stimulation, which increases the pharyngeal dilator muscle tone during sleep (Certal et al. 2015). On the other hand, a surgical technique that targets soft tissue elasticity in the upper airway includes palatal implants that increase the stiffness of the soft palate (Nordgård et al. 2004). However, the short-term and long-term success rate of these surgeries varies highly between patients, occasionally even resulting in a negative outcome (Sher et al. 1996; Carvalho et al. 2012).

Fundamentally, OSA is a mechanical problem where the negative pressure generated during inspiration exceeds the stresses generated by the surrounding soft tissues (via active and passive mechanical behavior) (Strohl et al. 2012). Therefore, the study of upper airway mechanics of collapse involving a combination of anatomical and non-anatomical features is of high interest. Clinical implementation of such a study requires substantial resources and can be very time-consuming. Numerical simulations on the other hand can often produce reliable outcomes with limited resources and time (Mielczarek and Uziałko-Mydlikowska 2012). Numerical simulations of upper airway fall into three categories: computational fluid dynamics (CFD) based (Aasgrav et al. 2016; Zhao et al. 2013a; Sung et al. 2006), finite element modeling (FEM) (Liu et al. 2018a; Henrik Strand Moxness et al. 2018; Huang et al. 2007; Lee et al. 2018; Amatoury et al. 2016), and fluid structure interaction (FSI) (Zhao et al. 2013b; Liu et al. 2018b); with each method having its own advantages and complications. CFD simulations analyze the airflow in both healthy and diseased human airway, where the airflow can be laminar, transitional, or turbulent. CFD simulations provide valuable insights on the complexity of airflow during breathing but only focus on the geometry and boundary conditions (Kharat et al. 2018), ignoring the soft tissues in the upper airway. FE simulations on the other hand focus on both passive (Henrik Strand Moxness et al. 2018) and active (Liu et al. 2019) mechanics of the soft tissues in the upper airway. The effect of airflow is modeled as a negative pressure gradient on the tissue surface and not included explicitly. FE simulations have shown great promise in aiding OSA treatment (Park et al. 2020). Nonetheless, the existing FE models are either limited to mechanics of the soft palate or exclude proper anatomical segmentation and thereby interaction between the main upper airway soft tissues: the palate, pharyngeal walls, tongue base, and the extrinsic muscles. FSI simulations on the other hand offer a more accurate representation of the problem with fluid flow (air) interacting with solid structures (soft tissues). However, setting up FSI simulations and bench-marking the results can be a cumbersome process, and the existing ones rely on gross simplifications of material modeling, boundary conditions, and/or coupling the two domains (Faizal et al. 2019).

Considering the recent advances in computational mechanics of upper airway (Pugachev et al. 2020; Caragiuli et al. 2021), patient-specific numerical models can be an interesting option for studying the influencing factors. We intend to develop patient-specific FE models of the upper airway including the palate, pharyngeal wall, tongue, and genioglossus muscle. The aim is then to test the impact of anatomic and physiologic surgical interventions such as Uvulopalatopharyngoplasty (UPPP), maxillo-mandibular advancement along with genioglossus advancement (MMA), palatal implants, and tongue implants on upper airway mechanics, with the ultimate goal of individualizing therapy on the basis of anatomic and physiologic measurements.

## Methods

We have developed a partial 3D anatomic model of the upper airway on the basis of computed tomography (CT) images from representative OSA patients ($n=5$). We define key points along the boundaries of all structures deemed essential for the model (tongue, mandible, hard palate, soft palate, uvula, and pharyngeal wall). Our major purpose is to test the effects of uvulopalatopharygoplasty (UPPP), maxillo-mandibular advancement along with genioglossus advancement (MMA), palatal implants ($Pillar^{TM} System, Restore Medical, St. Paul, MN$), and tongue implants on upper airway collapsibility in the anterior-posterior plane using the model.

**Fig. 1:**
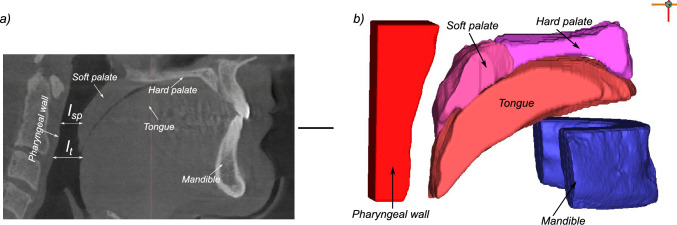
**a** Midsagittal section of the CT image scan of patient 1; **b** end result of the segmentation process in MIMICS, with the different tissues of interest marked separately

### Patient-specific geometry from CT data

Pre-operative CT scans of 5 patients suffering from OSA are used in this study. The study population is drafted upon an ongoing clinical evaluation of non-obese patients (BMI < 30), with no prior upper airway surgery and with no craniofacial abnormalities but with either a nasal blockage that required open functional rhinoplasty or an oropharyngeal crowdedness that required a lateral pharyngoplasty. In other words, the base of the tongue was deemed normal by physical examination, as was the neck circumference (men < 40 cm, women < 35 cm). The AHI was measured within 3 months prior to medical images. Images were taken in a decongested state (tetracaine 16 mg/ml and adrenaline 0,2 mg/ml - 5 ml in total). Usage of the images was approved by the Norwegian Regional Committee for Medical Research Ethics (REK) and was registered in clinicaltrials.gov (NCT01282125). The CT scans had a resolution of 0.46 mm in the anterior-posterior and lateral directions and 0.7 mm in the superior-inferior direction. For each patient, the minimal distance between the soft palate and the pharyngeal wall ($l_\text {sp}$) and the minimal distance between the tongue base and the pharyngeal wall ($l_\text {t}$) were measured. The patients’ demography and upper airway anatomy characteristics are reported in Table 1. The biomedical software Mimics (Materialize Mimics Innovation Suite, Mimics Research 19.0, Leuven, Belgium) was used to segment and post-process the sagittal, coronal, and axial CT scans and reconstruct them in 3D geometry. Briefly, the DICOM images as shown in Fig. 1a were imported to MIMICS for segmentation. A separate mask was created for each anatomical entity of interest, including soft palate, hard palate, pharyngeal wall, tongue, and mandible. Then, each of the above tissues were segmented through a semi-automatic region-fill algorithm, with user inputs provided when necessary to augment the segmentation process. The geometrical boundary between the tongue and the soft palate as well as the tongue and genioglossus muscle was made manually. The genioglossus muscle originates from the superior mental spine of the mandible and then, “fans” posteriorly to insert at the tip of the tongue and dorsum of the tongue (Silverstein et al. 2000; Sakamoto 2017). The direction of the fibers was assumed to be in the anterior-posterior direction ($\pm 2^{o}$). The end result of the segmentation process is shown in Fig. 1b. A partial 3D model was chosen with tissues segmented around the midsagittal plane with a width of up to 2.5 cm. This was done to keep the relevant soft tissues of the upper airway, owing to the blurry demarcation between the lateral walls and the extrinsic muscles in the CT scans. Secondly, unlike a 2d model, it enables us to study the collapse in the anterior-posterior direction more accurately given the heterogeneous distance map between the soft palate, tongue, and posterior pharyngeal wall.

**Table 1:** Patient demographics and measured geometrical parameters of interest

Patient	Age	Gender	BMI	AHI	$l_\text {sp}$ (mm)	$l_\text {t}$ (mm)
1	51	Male	29.1	15.5	1.3	1.7
2	44	Male	27.2	18.4	0.8	1.0
3	60	Male	24.1	25.2	0.9	1.3
4	51	Female	20.7	17.2	0.8	1.1
5	50	Female	26.5	13.7	0.5	1.0

*BMI* body mass index, $l_\text {sp}$ and $l_\text {t}$ are measured minimum distance between the pharyngeal wall and soft palate and tongue, respectively

### Finite element model

**Fig. 2:**
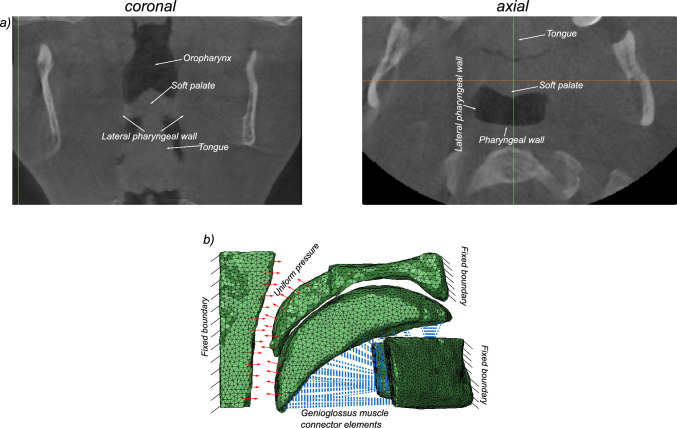
**a** The coronal and axial views of the upper airway showing the position and attachments of the soft tissues of interest; **b** boundary conditions applied on the patient specific finite element model. The posterior surface of the pharyngeal wall, the mandible, and the anterior surface of the hard palate are fixed boundaries. A ramp pressure loading is applied on the anterior surface of the pharyngeal wall, soft palate, and part of the tongue (red arrows), the genioglossus muscle is represented by connector elements (shown in blue)

#### Mesh generation

The tissues segmented as separate parts in MIMICS were then imported to ANSYS ICEM CFD^®^, a proprietary software package which provides advanced geometry/mesh generation and diagnostics. Each tissue geometry was independently meshed using ten node tetrahedral elements, with a maximum edge length of 0.7 mm. A minimum mesh quality of 0.8 was ensured using multiple smoothing iterations. Ten node tetrahedral elements (C3D10H) along with a nonlinear geometry toggle were used to mesh the tissues. The generated meshes of the individual tissue geometries were subsequently processed and integrated into a single input file for analysis in Abaqus^®^. The reconstructed finite element model and the applied boundary conditions are shown in Fig. 2b.

Mesh convergence studies have been made on the base model simulations with three different mesh densities in ICEM CFD (coarse, medium, and fine). The model was verified to be independent of mesh density. For the base models of all 5 patients, the deformation of the soft palate was compared. There was less than a 3.7% difference between the higher mesh densities and the current mesh (medium). The utilized mesh configuration had a minimum element size of 0.7 mm with 1360072 elements and 11882160 total DOF.

#### Material models and boundary conditions

The pharyngeal wall, soft palate, tongue, and genioglossus muscle were assumed to be fully deformable. The hard palate and the mandible were modeled as rigid bodies. Since the strain of the tongue and other pharyngeal tissues is small during the upper airway collapse (Huang et al. 2007), the passive tongue and all other pharyngeal tissues are assumed to be incompressible neo-Hookean hyperelastic materials. The strain energy function for an incompressible neo-Hookean solid reads.

1 $$\begin{aligned} \Psi = C_{1}(I_{1} - 3) \end{aligned}$$

where $C_{1}$ is a material constant, and $I_{1}$ is the first invariant (trace), of the right Cauchy-Green deformation tensor, i.e., $I_{1}$ = $\lambda _{1}^{2}$ + $\lambda _{2}^{2}$ + $\lambda _{3}^{2}$. $\lambda _{1}$, $\lambda _{2}$, $\lambda _{3}$ are the principle stretches. The equivalency with linear elasticity theory is established as:

2 $$\begin{aligned} C_{1} = \frac{E}{4(1 - \nu )} \end{aligned}$$

where *E* is the Young’s modulus, and $\nu$ is the Poisson’s ratio, which for an incompressible material is taken to be 0.5.

For the soft palate and the tongue, the material properties are derived from the literature (Haddad et al. 2018), which reported Young’s modulus of $6.54 \pm 3.52$ $\text {kPa}$ and $5.52 \pm 1.19$ $\text {kPa}$, respectively. Assuming a Poisson’s ratio value of 0.4999, the neo-Hookean material parameter was calculated for both tissues. The pharyngeal wall’s material parameter was set based on the modulus identified on tonsils ($E = 4.56 \pm 2.41$ $\text {kPa}$).

**Fig. 3:**
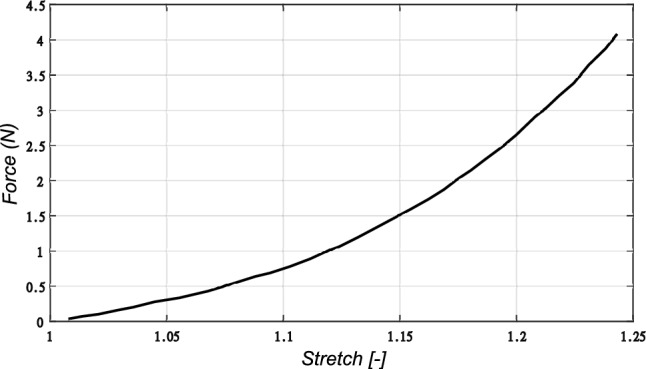
Nonlinear force-stretch response of the genioglossus muscle

The genioglossus muscle was modeled with 1-D connector elements arising from the mandible and inserted into the inferior surface of the tongue. The genioglossus is the most important muscle in upper airway collapse as it controls the protrusion of the tongue. The nonlinear force-length response of the muscle fibers (Yousefi et al. 2018) was directly input as tabular data in the connector section definition. Figure 3 shows the nonlinear force-length profile used for the muscle fibers.

The tongue is composed intrinsically of a complex network of longitudinal, transverse, and vertical muscle fibers (Sakamoto 2017). However, herein, it was assumed to behave passively as an isotropic hyperelastic material. The genioglossus muscle was assumed to be attached through the whole inferior surface of the tongue.

The hard palate, mandible, and posterior surface of the pharyngeal wall are considered fixed boundaries with zero displacements. The tongue, uvula, and other soft tissue, except the parts connecting directly to fixed boundaries, can move freely under loads in the anterior-posterior direction. An elastic boundary condition was applied in the lateral direction for the tongue and the uvula in order to simulate the presence of lateral muscles and the pharyngeal wall. The soft palate and the tongue have sliding movement on their contact surfaces during deformations. A general surface-surface contact constraint with frictionless behavior in the tangential direction and hard contact in the normal direction was applied. A uniform pressure loading is applied on the surface of the anterior pharyngeal wall, the soft palate, and the region of the tongue protruding outside the soft palate. For increasing pressure, the soft palate and the tongue deform gradually toward the pharyngeal wall. The first point of contact between either the soft palate or the tongue with the wall is considered to be the critical pressure for the onset of OSA.

### Anatomic and physiologic manipulations

**Fig. 4:**
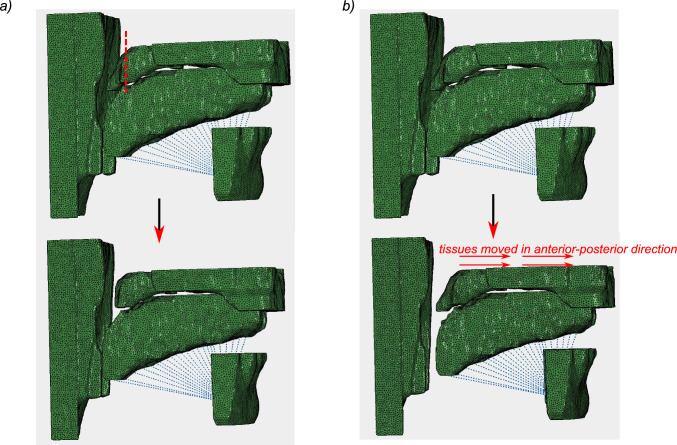
**a** Schematic of the UPPP surgical procedure, with a portion of the palate resected in the coronal plane; **b** representation of the maxillo-mandibular advancement surgery displacing the tissues forward

***Uvulopalatopharyngoplasty (UPPP)*** To investigate the effects of UPPP on upper airway collapsibility, we resected 50% of the uvula/soft palate from the segmented structure in the upper airway. We then simulated the deformation at different upper airway negative pressures to estimate the closing pressure (Pcrit) of the sleeping airway after uvula removal. A representation of the upper airway anatomy after resection of the soft palate is shown in Fig. 4a

***Maxillo-mandibular advancement*** In our model, we simulated maxillo-mandibular advancement simultaneously with genioglossus advancement by advancing the upper airway soft tissues and the mandible anteriorly by 1 cm as shown in Fig. 4b. We compared the calculated collapse of the upper airway without the advancement to compare the closing pressure of the upper airway with mandibular advancement.

Polyethylentherephthalate implants of Young’s modulus 244 $\text {MPa}$ as recommended in Nordgård et al. (2004) were inserted in the soft palate and the tongue (in two configurations).

**Fig. 5:**
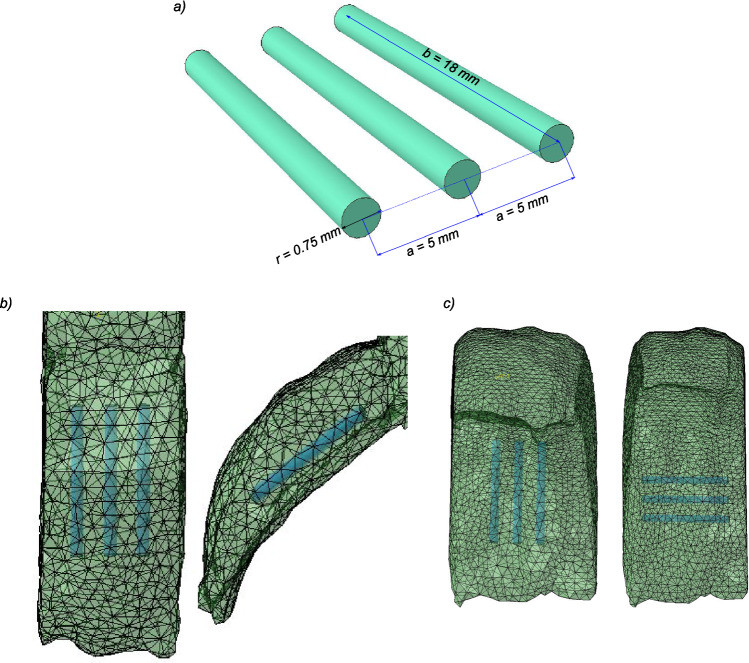
Schema of the physiologic manipulations using Polyethylentherephthalate implants (PET): **a** geometric characteristics of the PET implants, **b** schematic of the implant insertion in the soft palate, **c** the two schema of implant insertion in the tongue

***Palatal implants*** For palatal stiffening, we simulated the insertion of 3 palatal implants as reported in Nordgård et al. (2004). The implants had a length of 18 mm, a radius of 0.75 mm, and center to center separation of 5 mm. The elasticity of the palatal implants was previously determined by uniaxial tension testing in our group to be 244 MPa (Liu et al. 2018a), which agrees with the reported values on made of polyethylentherephthalate (PET) materials (Lam et al. 2003). The geometrical characteristics and schema of the implants is shown in Fig. 5a. The schematic of the insertion of the implants in the soft palate is shown in Fig. 5b. We chose a position for the implant based on the recommended placement site in Nordgård et al. (2004).

***Tongue implants*** Similar to the insertion of palatal implants, the same procedure was followed for tongue stiffening as this may represent a future method of treating OSA either alone or in combination with palatal implants. Two different insertion patterns were tested as shown in Fig. 5c. In each case, the implants were inserted at the tongue base perpendicular to the pressure vector. One longitudinal and one transverse alignment was chosen for implant placement to check the influence of tongue stiffening on OSA.

In both cases where the implants are inserted into the tissue, an embedded element constraint is used in Abaqus. The constraint allows the two structures to deform as one, with the total stiffness of the structure computed based on the rule of mixtures (Abaqus 2011).

### Statistical analysis

The one-way analysis of variance (ANOVA) was employed for statistical analysis to compare the UPPP, MMA, palatal implants, and tongue implants’ groups of specimens. The groups were significantly different if *p* $\le$ 0.05. Mean (± standard deviation), median, first and third quartile values (Q1, Q3) of the aforementioned groups were computed.

## Results

### Base model

**Fig. 6:**
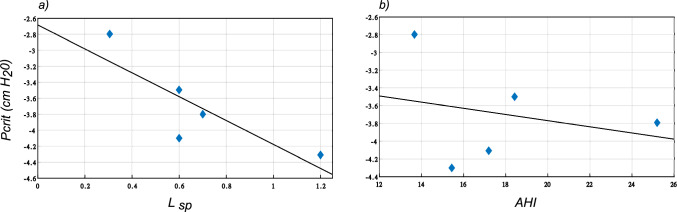
**a** The minimal aneterior-posterior distance between soft palate and the pharyngeal wall showed a correlation coefficient of $-$0.81 with Pcrit, with an $r^{2}$ of 0.63; **b** The correlation between AHI and Pcrit was $-$0.30, with an $r^{2}$ of 0.24

On the basis of the parameters outlined in Methods, we have performed a number of simulations to predict the mechanics of the upper airway. Denoting this as the base model without any anatomic or physiologic manipulations, the critical pressure for upper airway collapse was computed for each patient. In all cases, the first collapse occurred at the retro-palatal level, with the soft palate coming into contact with the pharyngeal wall. Based on our model results, the critical pressure varied between $-2.8$ and $-4.3$ $\text {cm}$ $\text{H}_{2}\text{O}$. In comparison with the minimum distance between the soft palate and the pharyngeal wall, the computed critical pressure showed a strong negative correlation (see Fig. 6a. A correlation coefficient of $-0.84$ and a goodness of fit measure, $r^{2}$ of 0.63 was recorded. This means that narrowing of the upper airway in the anterior-posterior plane has a significant impact on upper airway patency and leads to an easier collapse. On the other hand, only a weak negative correlation was observed between the closing pressure and patient’s apnea–hypopnea index (AHI) as shown in Fig. 6b. For this case, a correlation coefficient of $-0.30$ and a goodness of fit measure $r^{2}$ of 0.24 was recorded.

### Anatomic manipulations

**Fig. 7:**
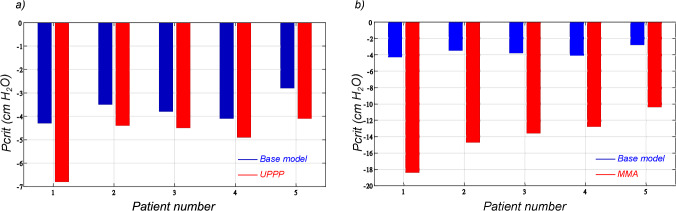
**a** Comparison between the critical pressure in the base configuration and after undergoing resection of the soft palate (UPPP); **b** Pronounced improvement in upper airway critical pressure after undergoing maxillo-mandibular advancement as compared to the base configuration

**Fig. 8:**
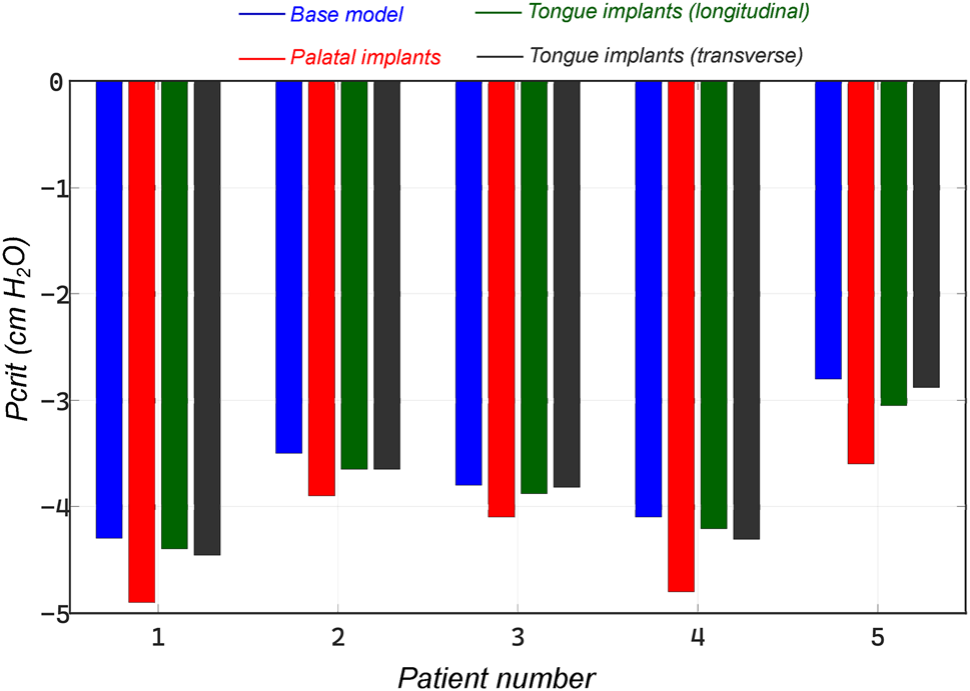
Influence of surgical palatal implants for soft palate stiffening on the critical pressure, and increasing stiffness of the tongue base region only leads to minimal improvement in upper airway critical pressure

**Fig. 9:**
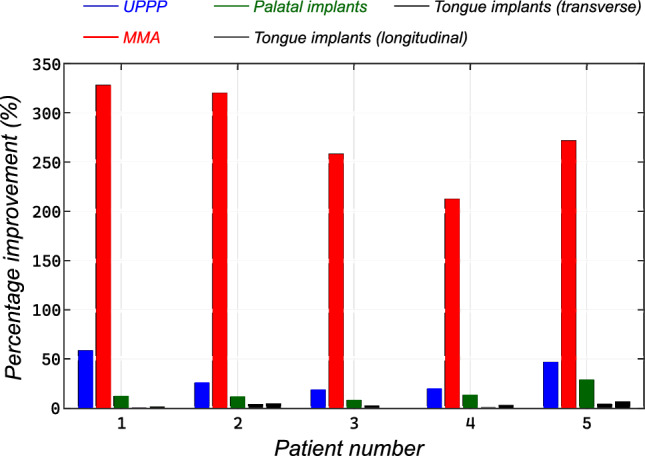
Comparison of improvement in upper airway collapse pressure among different physiological and anatomic manipulations with respect to the base model

All of the anatomic manipulations led to notable decreases in the collapsibility of the upper airway. As can be seen in Figs. 7, 8, 9, progressively more negative airway pressures were necessary to occlude the pharyngeal airway for both tested surgical procedures.

***Uvulopalatopharyngoplasty (UPPP)*** Excision of a portion of the soft palate and the uvula changes the flow pattern and pressure distribution in the pharyngeal airway substantially and, therefore, affects upper airway collapsibility. Figure 7 shows the improvement of upper airway collapsibility after being subjected to UPPP and MMA. For instance, patient 1 showed the most improvement of $58.13 \%$ from $-4.3$ to $-6.8$ $\text {cm}$ $\text{H}_{2}\text{O}$. Once sufficient palate has been resected, occlusion occurs behind the tongue at the retro-lingual level, with no further improvements in the mechanics occurring with greater palate resection. A minimum improvement of $19.12 \%$ was observed from the results as shown in Table 2. This indicates the influence of the length of the soft palate and the efficacy of UPPP as a measure to treat OSA.

***Maxillo-mandibular advancement*** Mandibular advancement showed the most pronounced improvement in terms of critical pressure with a minimum of $212.2 \%$ in patient 4. The normal airway closes at $-4.1$ $\text {cm}$ $\text{H}_{2}\text{O}$ pressure but remains patent at this pressure once the mandible and hard palate are advanced by 1 cm. The airway did not actually close until $-12.8$ $\text {cm}$ $\text{H}_{2}\text{O}$ is applied to the pharyngeal airway with an advanced mandible and hard palate. The largest improvement in critical pressure of $327.91 \%$ was seen in patient 1.

**Table 2:** Computed critical pressure for all patients in the base configuration and after undergoing UPPP and MMA surgeries

Patient	$P_\text {crit}$ (base)	$P_{\text {crit}}$ (UPPP)	$\Delta$ (UPPP)	$P_{\text {crit}}$ (MMA)	$\Delta$ (MMA)
1	$-$4.3	$-$6.8	58.14%	$-$18.4	327.91%
2	$-$3.5	$-$4.4	25.71%	$-$14.8	320.05%
3	$-$3.8	$-$4.9	28.95%	$-$13.6	257.89%
4	$-$4.1	$-$4.9	19.12%	$-$12.8	212.20%
5	$-$2.8	$-$4.1	46.41%	$-$10.4	271.42%

All the reported pressure values are in $\text {cm}$ $\text {H}_{2}\text {O}$

The computed values for critical pressure in the base configuration, after UPPP, and after MMA procedures are presented in Table 2.

### Physiologic manipulations

***Palatal implants*** As can be seen in Table 3 and Fig. 8, the palatal implants had a substantial impact on improving the upper airway mechanics. In all cases, the improvement in critical pressure was less than the one computed from UPPP surgery which removes a section of the soft palate. Moreover, the two groups of results were found be to significantly statistically different ($p-\text{value} = 0.0024$). This magnitude of the effect is on the order of what we have previously observed comparing normal men to normal women, suggesting the observed findings are clinically significant (Malhotra et al. 2002). Patient 4 had an improvement of $12.07\%$ in critical pressure, which was notably similar to the improvement found after UPPP surgery. Overall, patient 5 had the best improvement in terms of critical pressure which improved from $-2.8$ $\text {cm}$ $\text{H}_{2}\text{O}$ to $-3.6$ $\text {cm}$ $\text{H}_{2}\text{O}$.

***Tongue implants*** Here, we assessed the role of tongue stiffening in the base region, which is exposed to the negative pressure developed during inspiration. Also, in Fig. 8, we explored the effect on upper airway collapse in reaction to these mechanical manipulations. Neither of the two tongue implants’ schemes showed any significant improvement in terms of critical pressure. A meager improvement of $3.9\%$ and $6.4\%$ was seen in patient 5 as compared to $28.57\%$ after UPPP. The rest of the cases had a similar characteristic with minor improvement in critical pressure between $0.5\%$ and $6.4\%$. The groups were not significantly different ($p-\text{value} = 0.0007$). These values reflect an airway less collapsible but not a significant improvement to consider it a benefit to airway stability.

**Table 3:** Computed critical pressure for all patients in after insertion of palatal and tongue implants

Patient	$P_{\text {crit}}$ (PI)	$\Delta$ (PI)	$P_{\text {crit}}$ ($TI_{1}$)	$\Delta$ ($TI_{1}$)	$P_{\text {crit}}$ ($TI_{2}$)	$\Delta$ ($TI_{2}$)
1	$-$4.8	11.65%	$-$4.34	0.9%	$-$4.37	1.6%
2	$-$3.9	11.43%	$-$3.63	3.7%	$-$3.65	4.3%
3	$-$4.1	7.89%	$-$3.88	2.1%	$-$3.82	0.5%
4	$-$4.6	12.07%	$-$4.15	1.2%	$-$4.21	2.7%
5	$-$3.6	28.57%	$-$2.91	3.9%	$-$2.98	6.4%

All the reported pressure values are in $\text {cm}$ $\text{H}_{2}\text{O}$.

*PI* palatal implants, $TI_{1}$ tongue implants in longitudinal configuration, $TI_{2}$ tongue implants in transverse configuration

### Statistics

On average maxillo-mandibular advancement (MMA) predicted an improvement of Pcrit by 277.4% (± 47.5), uvulopalatopharyngoplasty showed also a significant improvement of Pcrit by 35.4% (± 16.2). Following the one-way ANOVA testing, the two groups were found to be significantly different from each other (*p*-value < 0.05). Palatal implants showed an improvement in Pcrit by themselves, with a mean of 14% (± 7.8). The group was found to be statistically different from both UPPP and MMA (*p*-value < 0.05). Tongue implants in both longitudinal (2.4% ± 1.4) and transverse (3.2% ± 2.1) directions showed minimal improvement in Pcrit, while the two groups were not statistically different from one another (*p*-value 0.05). It is clearly depicted in Fig. 9 that MMA is an effective treatment for improvement of critical closing pressure.

## Discussion

In this study, we demonstrate a novel patient-specific partial 3D finite element model of the upper airway that is based on pre-operative CT scan images and designed to simulate the effects of airway deformation under applied intraluminal pressures. The presented model includes relevant anatomical features including the soft palate, tongue, genioglossus muscle, and pharyngeal wall. As applications of the developed model of the upper airway, we also simulated the effects of two anatomic manipulations (i.e., uvulopalatopharyngoplasty and maxillo-mandibular advancement) and the effects of two physiologic manipulations (i.e., palatal implants and tongue implants) on the mechanical conditions of the upper airway. All the manipulations that are to be considered as possible surgical methods showed a marked improvement in the critical pressure at collapse, and we can assume that these results are consistent even with $n>5$. In particular, the maxillo-mandibular advancement showed the greatest improvement, followed by palatal resection, palatal implants, and finally tongue implants. The main novelty of the study is the inclusion of the tongue and its interaction with the soft palate during breathing. We believe that the present study provides proof of concept that upper airway surgical therapy can be individualized using available imaging and computational techniques. This strategy may be useful in the future to optimize therapeutic strategies but will obviously have to be tested in real patients undergoing surgery.

The success rate associated with various surgical treatments for OSA have been highly variable (Carvalho et al. 2012). Several ethical issues and other challenges (Brown et al. 2011) hinder the possibility of a robust clinical trial in this area. Further, the high variability in surgical treatments makes it difficult to perform comparative studies. Thus, despite excellent attempts at clinical research in the area of upper airway surgery, progress has been somewhat slow. The role of computational modeling techniques and digital twins has shown promising developments as diagnostic tools for other conditions (Hemmler et al. 2019; Derycke et al. 2020).

Although further model validation is necessary some observations indicate that the results from our model do indeed predict reality. With regard to the UPPP surgery, the measured improvement in upper airway critical pressure is in line with reported values (De Vito et al. 2021). Based on a 2d computational model (midsagittal plane) of normal subjects, Huang et al. (2005) reported an improvement of 38.4% (− 13 cm $\text {H}\_2\text {O}$ to − 18 cm $\text {H}\_2\text {O}$). This is equivalent to the mean improvement of 35.4% ± 16.2% observed in our model. It is worthy to note that the computations were made on a mean upper airway geometry composed of normal adults. A similar partial 3D model (Huang et al. 2007) based on MRI images of OSA patients produced an improvement of 155% where much of the palate had been resected, while two intermediate progressively resectioned configurations produced 20% and 60% improvements, respectively. The choice of $1 \text {cm}$ for maxillo-mandibular advancement is in line with reported surgical planning records (Susarla et al. 2011; Van der Cruyssen et al. 2019). Although clinical reports on the improvement of critical pressure are somewhat sparse, the observed improvement in our model is in good agreement with the widely reported high success rate (Zaghi et al. 2016; Holty and Guilleminault 2010) of the surgery. However, the developed 2d model of Huang et al. (2005) has produced a meagre improvement of 61% compared to 277% in our model. Amatoury et al. (2016) developed a detailed 2d FE model of the rabbit upper airway and conducted simulations on mandibular advancement which were validated against experimental investigations. Their model provides evidence for soft palate stiffening following MMA and further reinforces the role of hyoid bone in load transfer after the procedure. In our normal airway model, we also assessed the effects of palatal implants on pharyngeal mechanics and predicted changes of similar magnitude to those seen for other commonly performed upper airway procedures, where our findings are again in keeping with the available clinical data (Choi et al. 2013). While the 2d model developed by Huang et al. (2005) had an improvement of 46%, the partial 3D model based on OSA patients’ geometry predicted an average improvement of 20.8% compared to our model prediction in $\delta$Pcrit by 14%. It should be noted that since the critical pressure values in normal subjects are somewhat variable in the literature, our simulations are most useful for examining the relative changes in critical pressure measures, since the absolute values will depend greatly on the individual patient. Along these lines, we believe that our model is effective in predicting reality but acknowledge that ongoing efforts are needed in this area.

**Fig. 10:**
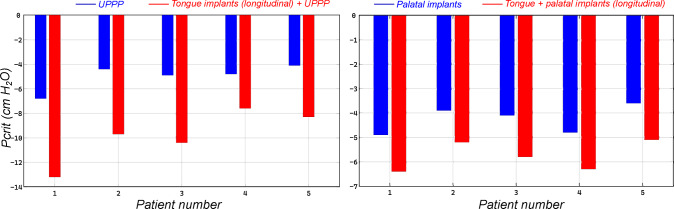
left: Significant improvement in upper airway mechanics via tongue stiffening after UPPP surgery; right: Similar incremental improvement in airway critical pressure when both palatal and tongue implants are simultaneously deployed

The role of the tongue in upper airway mechanics and OSA is widely accepted. The position of the tongue (Zhao et al. 2020), the size of the tongue at its base (Lahav et al. 2009), and excessive fat depositions at the tongue base (Kim et al. 2014) were all found to be associated with the severity of OSA. However, it is often neglected in computational models when assessing OSA. The strategy of tongue stiffening to treat sleep apnea has received minimal attention in the literature. Some authors have argued that the ineffectiveness of isolated palatal procedures may reflect ongoing compromise in the retro-lingual airway. Similarly, isolated tongue procedures are unlikely to work if anatomic compromise persists in the retro-palatal airway (Millman et al. 1998). From our simulations, we see that the interaction of the tongue with the soft palate can be important in determining the upper airway collapse in some patients with a constricted airway. For instance, when tongue implants were used in either the longitudinal or transverse direction, a marginal improvement was seen in critical pressure. This could be due to a reduction in the pressure exerted by the tongue on the soft palate, further pushing it to collapse in addition to the developed negative pressure. Secondly, further investigating the UPPP surgery as a treatment method, combining it together with tongue implants, has shown added improvement almost comparable to the level of MMA surgery. However, the difference between longitudinal and transverse schemes was not found to be significantly different. Finally, the simultaneous deployment of palatal and tongue implants also showed a minor improvement in critical closing pressure compared to only palatal or tongue implants. The computed values of critical pressure in these two cases are shown in Fig. 10. Therefore, it could be proposed that tongue stiffening could be beneficial for patients who failed to benefit from UPPP and palatal implants.

A conscious decision was made to use a partial 3D model in order to include individual tissue structures and compute the mechanics of upper airway collapse primarily in the anterior-posterior direction. It should be noted that the mechanics of upper airway collapse is multi-dimensional and could occur also due to narrowing (Liu et al. 2007) or loss of soft tissue mechanical function (Andrade da Silva Dantas et al. 2012) in the lateral pharyngeal walls. Although the anatomical aspect of the lateral pharyngeal wall could not be assessed in this study, the influence of its elasticity was studied. For this, on a single geometry, the lateral surfaces of the soft palate were imposed with an elastic boundary condition with varying stiffness (from low stiffness to almost rigid) in the lateral direction. The results from this analysis showed that the influence was indeed negligible. However, we think that this could be due to a bias in the loading protocol which fundamentally deforms the tissue in the anterior–posterior direction. Moreover, multiple studies have shown that pharyngeal narrowing primarily occurs in the anterior-posterior direction (Horner et al. 1989). In any case, future studies should be aimed at including the full 3D anatomy of the upper airway structures and their interactions.

**Fig. 11:**
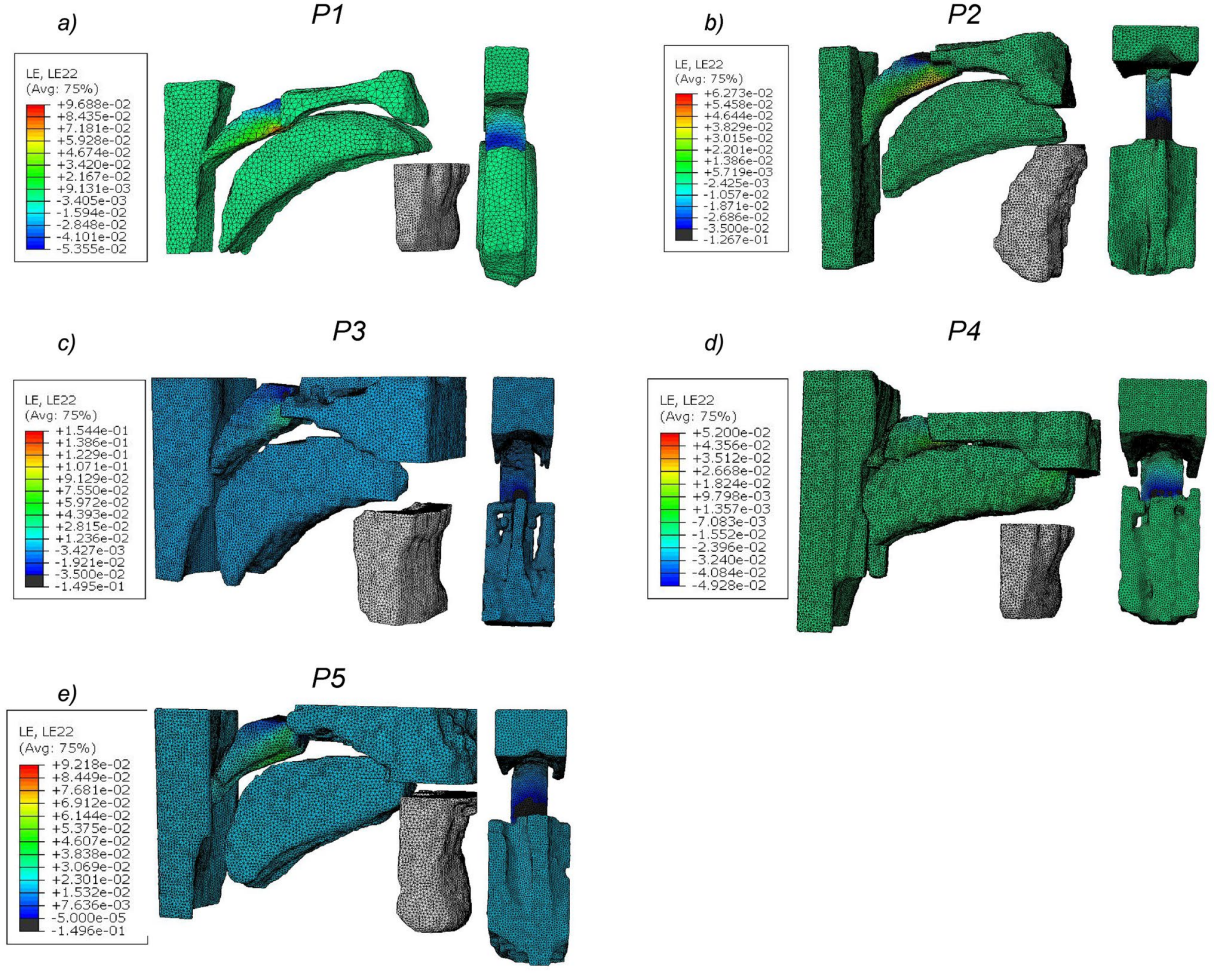
**a**–**e** Transverse strain in the tissues of the upper airway at closing pressure in all patients. On the left in each sub-figure is the sagittal view and on the right is the superior view of the logarithmic strain in each patient-specific finite element model

**Fig. 12:**
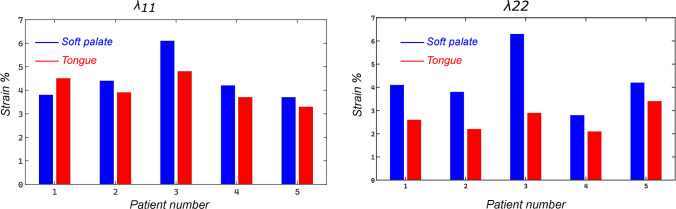
The computed absolute average strain over all elements within the soft palate and the tongue in **a** anterior-posterior direction; **b** transverse direction

Despite its strengths and important insights, the study has a few limitations. Firstly, the material models for the soft tissues were assumed to be isotropic neo-Hookean hyperelastic. In reality, the response of soft tissues in complex mechanical environments is often nonlinear and anisotropic due to inherent variations in underlying microstructural constituents (Strohl et al. 2012; Bilston and Gandevia 2014). An ongoing experimental study on testing soft tissues from human upper airways (n=1) does confirm this (see appendix A).Fig. 11a–e shows the transverse strain levels in the soft palate and the tongue at closing pressure in the base configuration. A similar trend was observed in all patients for the longitudinal strain. As can be seen, the average strain in the model at closing stays around 4–6% in both components (see Fig. 12). It is to be noted that both strain components are higher in the transition zone between the hard palate and the soft palate, due to a shift from a very stiff material to soft tissue. The magnitude of strain remains sufficiently low in the posterior zone where the tissues collapse into the pharyngeal wall. Direct comparisons can then be made at this point to compute the deviation between the assumed neo-Hookean model and the actual anisotropic material response. The error levels and the difference between the two models are shown in Appendix A. For a strain level of up to 5%, a small error was observed between the neo-Hookean and the recorded material response. However, regional variations in material properties were recorded. One possible hypothesis to be investigated based on this would be to evaluate regional variations in material stiffness in patients suffering from OSA in comparison with healthy subjects to get a better understanding of the role of soft tissue passive mechanical properties. We are currently working on testing more upper airway tissues from human donors, in order to define anisotropic material properties to be used in more accurate simulations.

Further, given that the utilized parameters were derived from supposedly healthy subjects, one could argue that its importance on upper airway surgery is of minimal importance. However, in part, the use of patient-specific geometries enables us to assess the importance of anatomic manipulations on upper airway collapse. Using this as a benchmark, material data from OSA patients with varying degrees of severity could be included. Thus, despite this limitation, we believe our results both provide physiologic insights and do have clinical relevance.

The role of the anatomic position of the hyoid bone in effective load transfer and maintaining upper airway patency has been well demonstrated (Janicka and Halczy-Kowalik 2006). Pharyngeal constrictor and hyoglossus muscles, which are the superior muscle attachments to the hyoid also play an important role in upper airway mechanics (Knaack and Podszus 1998). These anatomic structures have not been included in the modeling process. The upper airway is an amalgamation of several muscles that together control the various functions involved (breathing, swallowing, speaking, etc.). With regard to upper airway collapse in OSA in particular, the muscle tensor veli palatini and the genioglossus muscles have been reported to play an important role (McWhorter et al. 1999; Mediano et al. 2019). Several reports suggested that pharyngeal dilator muscles experience reduced tonic and phasic activation in OSA patients as compared to healthy subjects (Malhotra et al. 2000). Although the genioglossus muscle was modeled using connector elements with nonlinear stiffness to reflect its passive behavior, other muscle groups and their active response are overlooked in this study, but should be included in future studies. One reason for this was that although phenomenological models for active muscle response exist, experimental data on airway skeletal muscle are not known to the best of our knowledge. A sensitivity analysis on the influence of genioglossus muscle stiffness (force) on upper airway collapse however showed that it is important in restricting the tongue from moving in the posterior direction. It is believed that hypoglossal nerve stimulant therefore could also be beneficial for patients who failed to benefit from UPPP and palatal implants.

Although patient-specific geometries were used in developing the FE models, not all upper airway soft tissues were segmented on the model development process. The lack of lateral walls and extrinsic muscles of the tongue and the soft palate does not accurately represent the problem. This could in turn lead to over-estimation of the collapsibility of the upper airway. The use of elastic boundary conditions for the lateral direction might not fully replicate the intricate 3D anatomy of the upper airway. Secondly, actual breathing was not simulated in the model, but rather a ramp loading of negative pressure was applied on the soft tissues to estimate the critical closing pressure. For a better representation, patient-specific breathing profiles should be used to understand the influencing factors of collapse. Finally, as stated in the introduction, the problem posed in the mechanics of upper airway involves both fluid (airflow) and solid domains (soft tissue deformations). Although meaningful insights could be derived from the current finite element simulations, there is a pressing need for more robust analysis of upper airway mechanics. For instance, the first point of contact in the simulations might not necessarily indicate critical closing pressure, as there could be air flowing through the lateral sides of the upper airway space. It was assumed for the sake of modeling that it is representative of critical closing pressure in this study. In this regard, our current model should be extended to capture the necessary fluid–structure interactions that occur in the upper airway.

## Conclusion

In conclusion, we believe that computational modeling performed on patient specific medical imaging data, reinforced with gross physiological aspects of OSA patients could be a viable approach to predicting the likelihood of surgical success for the treatment of sleep apnea. Our model suggests that maxillo-mandibular advancement produces the best result in terms of improving critical pressure for upper airway collapse. Since it is only recommended for severe OSA patients, our model results suggest that patients who do not benefit from solely UPPP or palatal implants could benefit from simultaneous targeting of both retro-palatal and retro-lingual airway space. Thus, indicating a possible interaction between multiple mechanisms leading to upper airway collapse. Further investigation will be required to determine the clinical relevance of these observations in OSA patients. Such a model could be molded toward a pre-operative diagnostic tool for OSA surgery, but the validation of these models in larger-scale studies is needed prior to widespread use in surgical decision making.

## Figures and Tables

**Fig. 13:**
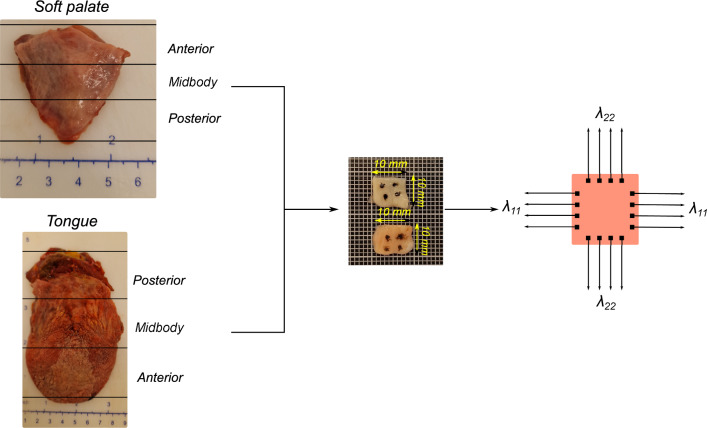
Excised human soft palate and tongue tissues following an autopsy; representative square specimens prepared for mechanical testing; and a schema of the biaxial testing protocol

**Fig. 14:**
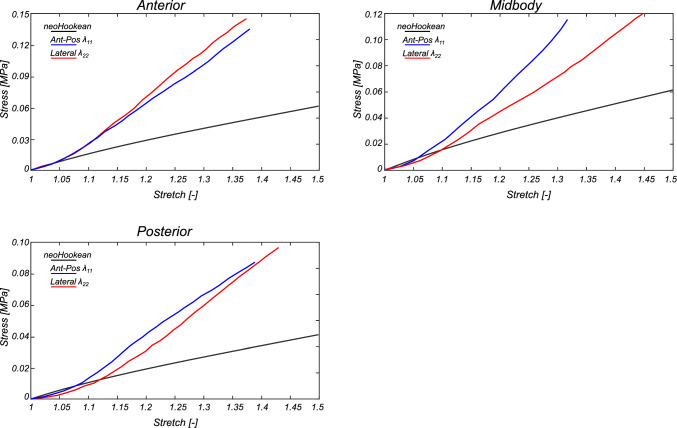
Regional variations in the nonlinear anisotropic mechanical response of the human soft palate

**Fig. 15:**
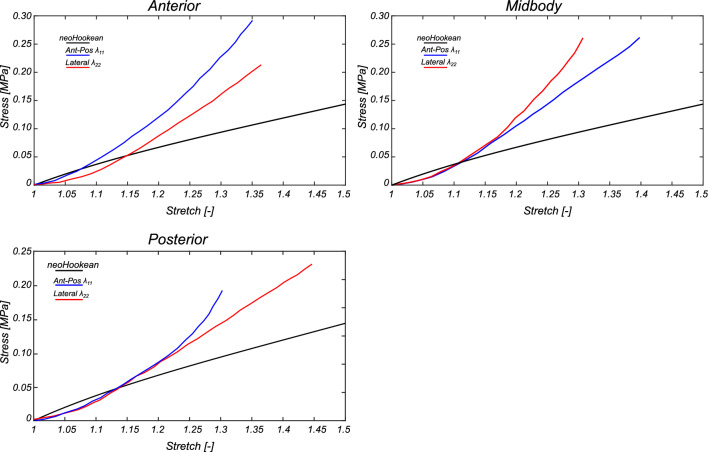
Regional variations in the nonlinear anisotropic mechanical response of the human tongue

## Appendix A: Material anisotropy of upper airway soft tissues

Soft tissues in the upper airway manifest nonlinear and anisotropic material behavior in response to mechanical stimulus. The regional variation of the mechanical response of the human soft palate tissue and tongue is shown here.

*Testing protocol* Upper airway soft tissues including the soft palate and the tongue were excised from a postmortem autopsy ($n=1$) conducted by a pathologist at St Olav’s hospital, Trondheim. The collection and use of the tissues for research are approved by the regional ethics committee. After excision, the samples were carefully transported to the biomechanics laboratory and stored in a freezer operating at $-28^{o}C$. All tissues were tested within 2 months of procuring them in adherence to the regional ethics committee rules for handling the tissue.

For the testing part, to prepare the specimens for the test, they were first thawed at $4^{o}C$ and then at room temperature in a phosphate buffer saline (1$\times$PBS) solution. Square specimens of length 10 mm were cut out from anterior, midbody, and posterior regions of the tissue as shown in Fig 13. The sample thickness is then measured three times with a caliper (0.01 mm resolution), with the average value used for post-processing the raw data. A tissue marking dye (Leica Biosystems, Germany) is used to mark the tissue in the center covering an area of approximately $2\,\text{mm}$ $\times$ $2\,\text{mm}$. The specimen is then mounted on to the biaxial test rig, which has been previously validated, see Sadeghinia et al. (2022), using surgical Gore-Tex CV5 sutures (Gore Medicals, USA) and barbless hooks. The sample is ensured to be submerged in a 1$\times$PBS bath at $37^{o}C$ throughout the duration of the test. Four step motors individually control the applied displacement via inductive displacement sensors (BAW M18MG, Balluff, Germany). The force generated is measured using an axial load transducer (U9C/50 HBM, Germany). Three different displacement controlled tests were conducted with one equi-biaxial loading, and two other sets with proportional loading. The two orthogonal directions were ensured to represent anterior-posterior and lateral alignment of the tissue. The cauchy-stress versus stretch responses were computed from the recorded force-displacement measurements and reported below. See Sadeghinia et al. (2022); Ayyalasomayajula and Skallerud (2022) for more details of the test setup.

*Mechanical response* The resulting stress-stretch curves for the soft palate specimens from anterior, midbody, and posterior regions are shown in Fig. 14. It can be observed that all stresses appear to increase with increasing strain. Interestingly, the soft palate showed an anisotropic mechanical response of varying intensity within each region. While the anterior-posterior direction was stiffer in the middle, the difference in stiffness was not significant in the other two regions. At the same strain, the anterior region showed the largest stress (in both orthogonal directions), followed by the midbody, and finally the posterior region.

The resulting stress-stretch curves for the tongue specimens from anterior, midbody, and posterior regions are shown in Fig. 15. A similar exponential profile of stress with increasing strain was observed in all specimens of the tongue tissue. With regard to regional differences, the anterior and midbody specimens showed similar values of stress at a given strain. In contrast, the mechanical properties of the tongue’s posterior region showed clear differences with the other two regions, with the stress in the posterior specimen found to be lower than the other two regions. The specimens from all regions also showed varying intensities of anisotropy, with the lateral direction being stiffer in midbody, and the anterior-posterior direction being stiffer in the other two.

Comparing the two tissues, the stress measured in the specimens from the tongue is approximately two times more than those of the soft palate under the same strain conditions. For instance, at a stretch of $\lambda = 1.20$, the average stresses in the tongue and soft palate are $110 \pm 11.2$ kPa $650 \pm 13.7$ kPa, respectively.

*Remarks* The isotropic neo-Hookean material model used for soft palate and tongue is represented by the black curve in Figs. 14 and 15. The corresponding error in using an isotropic hyperelastic model in place of a anisotropic hyperelastic constitutive model was evaluated. As shown in Fig. 12, the maximum transverse and longitudinal strains in both tissues largely do not exceed 5%. With this as a reference value, the corresponding error for soft palate specimens was $1\%$, $4.3\%$, and $5.6\%$, respectively, for anterior, midbody, and posterior regions. Similarly, with increasing strains of up to 12.5%, the corresponding error increased to $27.3\%$, $16.2\%$, and $14.5\%$ respectively for anterior, midbody, and posterior regions.

Within the tongue, a similar trend was observed. Up to a strain of 5%, the corresponding error for tongue specimens was $3.7\%$, $11.2\%$, and $9.4\%$, respectively, for anterior, midbody, and posterior regions. For a strain value of up to 12.5%, the error increased to $23.2\%$, $21.3\%$, and $20.6\%$, respectively, for anterior, midbody, and posterior regions.

It is important to note that an anisotropic material model will produce a more realistic representation of the upper airway soft tissue’s mechanical behavior. However, given the low number of tested specimens and given that the error is within acceptable range for the strains recorded in the simulations, the neo-Hookean model was chosen as an appropriate approximation.
